# Dispersion-type Hall resistance in InSb|Pt hybrid systems

**DOI:** 10.1038/srep22085

**Published:** 2016-02-24

**Authors:** Y. Shiomi, E. Saitoh

**Affiliations:** 1 Institute for Materials Research, Tohoku University, Sendai 980-8577, Japan; 2 Spin Quantum Rectification Project, ERATO, Japan Science and Technology Agency, Aoba-ku, Sendai 980-8577, Japan; 3 WPI Advanced Institute for Materials Research, Tohoku University, Sendai 980-8577, Japan; 4 Advanced Science Research Center, Japan Atomic Energy Agency, Tokai 319-1195, Japan

## Abstract

In nonmagnetic semiconductors and metals, most of Hall resistance exhibits a linear dependence with applied magnetic fields. In this work, by combining conduction in a metal and a semiconductor under external magnetic fields, we realize a dispersion-type magnetic-field dependence of Hall resistance. The dispersion-type Hall resistance appears in a broad temperature range below 150 K, where quantum linear magnetoresistance is noticeable in the semiconductor substrate. This unconventional Hall response in metal|semiconductor hybrid systems is explained by a change in dominant conduction from the semiconductor to the metal with increasing magnetic field strength.

Electric conduction is a most fundamental physical phenomenon in materials. According to the property of electric conduction, conducting materials are classified into semiconductors and metals. In semiconductors, the Fermi level is located in a band gap, while, in metals, it is in an energy band. This difference significantly affects conduction properties of metals and semiconductors. In general, electric conduction is characterized by electric resistance, which depends on carrier densities and mobilities. Since electric carriers are produced by thermal excitation and doping in semiconductors, carrier densities of semiconductors change significantly with temperature, while those are almost constant with temperature in metals. Mobilities, by contrast, increase with decreasing temperature both in semiconductors and metals, because scattering events occur less frequently at lower temperatures. Electric resistances of typical semiconductors increase exponentially with decreasing temperature because of reduced carrier densities, but in some semiconductors with small effective masses of conduction electrons, *e.g.* indium antimonide (InSb), the resistance often shows a complex temperature dependence, depending on the temperature dependences of carrier densities and mobilities^1^.

Responses of electric conduction to external magnetic fields in semiconductors are also different from those in metals. When conducting materials are subjected to external magnetic fields, the trajectory of moving electrons is curved by the Lorentz force. This deflection causes a Hall effect. Since Hall resistances are proportional to the inverse of the carrier density, the magnitudes of Hall resistances are large and sensitive to temperature in semiconductors, while Hall resistances are very small and almost constant with temperature for metals. Also, the Lorentz force affects electric resistance, measured in the parallel direction to the electric current. Because of the deflection of electrons’ motion, electric resistance becomes greater at higher magnetic fields. This is a main cause of the positive magnetoresistance observed in some semiconductors and metals. Since the magnitude of the magnetoresistance depends on mobilities, magnetoresistance is typically small in metals, while large magnetoresistance is often observed in clean semiconductors with high mobilities. Semiclassically, the magnetoresistance increases quadratically with magnetic fields, and then saturates at strong magnetic fields. In slightly inhomogeneous semiconductors, silver chalcogenides Ag_2+*δ*_Se and Ag_2+*δ*_Te^2^ and InSb^3^, on the other hand, giant linear magnetoresistance was reported. Abrikosov showed that this linear magnetoresistance is explained by a quantum effect (quantum linear magnetoresistance) on the assumption that all the electrons occupy the lowest Landau level under strong magnetic fields^4^,^5^,^6^.

In the present manuscript, we report magnetoresistance and Hall effects in a semiconductor|metal hybrid system. The sample structure is illustrated in Fig. 1(a). A polycrystalline Pt film is formed on an electron-doped InSb singlecrystalline substrate. InSb is a narrow gap semiconductor with low carrier concentration, small conduction electron effective mass, large Fermi wavelength, and extremely long carrier mean free path^7^. Because of these unusual electronic properties, InSb has very large magnetoresistance and Hall effects^7^,^8^,^9^,^10^,^11^. In fact, InSb typically has been used in Hall sensors. In the Pt film, by contrast, the magnetoresistance and Hall effects are very small, whose magnitudes are almost negligible compared with those of InSb. By combining the two materials with totally different conduction properties, we have observed unconventional magnetic-field profiles of magnetoresistance and Hall resistance in InSb|Pt at low temperatures where the quantum linear magnetoresistance is observed in the InSb substrate.

## Results

**Figure 1:**
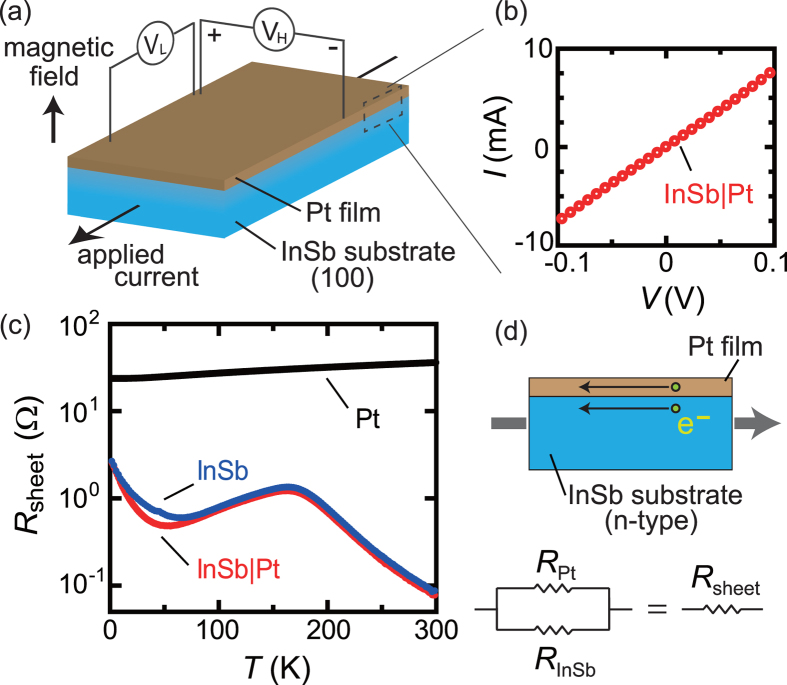
Experimental setup and sample properties. (**a**) Schematic illustrations of the sample structure and the experimental setup. (**b**) A current-voltage curve for an InSb|Pt sample. (**c**) Temperature (*T*) dependence of the sheet resistance 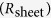 for InSb, InSb|Pt, and oxidized-Si|Pt. (**d**) An equivalent circuit for electric conduction of InSb|Pt samples.

Figure 1(b) shows a current-voltage (*I*-*V*) curve for an InSb|Pt sample. Here, the *I*-*V* curve was measured across the InSb|Pt sample. As shown in Fig. 1(b), the linear dependence is clearly observed, which supports the ohmic contact between the InSb substrate and the Pt film.

The sheet resistance 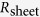 for InSb, InSb|Pt, and oxidized-Si|Pt (hereafter denoted just by Pt) is shown as a function of temperature, *T*, in Fig. 1(c). The sheet resistance of Pt is almost ~40 Ω at 300 K corresponding to the resistivity of 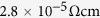, and slightly decreases with decreasing temperature. The weak temperature dependence in the polycrystalline Pt film indicates that the mobility of Pt hardly changes with temperature. The sheet resistance of InSb is, by contrast, much smaller than that of Pt in the entire temperature range, as shown in Fig. 1 (c). The sheet resistance of InSb is ~0.1 Ω at room temperature and increases with decreasing temperature from 300 K. Below about 150 K, the sheet resistance of InSb begins to decrease with decreasing temperature, and then increases again at low temperatures below 50 K; similar non-monotonic temperature dependence for InSb single crystals was also reported before^3^,^12^. As shown in Fig. 1(c), the sheet resistance of InSb|Pt almost coincides with that of InSb in the whole temperature regime. This means that almost all the electric current passes through the InSb layer under the zero magnetic field, because the magnitude of the sheet resistance of Pt is more than ten times greater than that of InSb.

**Figure 2:**
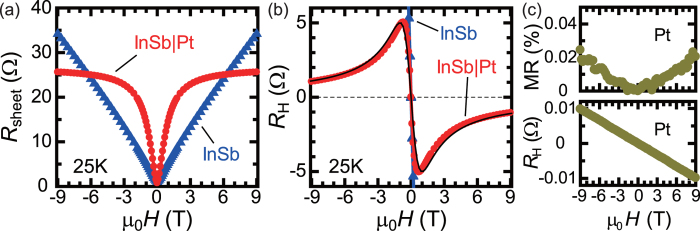
Magnetoresistance and Hall resistance at 25 K. Magnetic field (*H*) dependence of (**a**) the sheet resistance 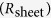 and (**b**) the Hall resistance 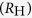 for InSb (triangle, blue) and InSb|Pt (circle, red). For reference, a dispersion-type function, 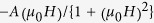 (*A*: constant), is shown in (**b**) (black curve). (**c**) Magnetic field (*H*) dependence of the magnetoresistance (MR) ratio 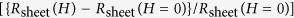 and the Hall resistance 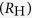 for Pt. Their magnitudes for Pt are negligibly small compared with those for InSb.

Without external magnetic fields, applied electric currents almost flow through the InSb layer, as shown in Fig. 1(c). This situation, however, changes dramatically, when the sample is subjected to external magnetic fields. Figure 2(a) shows the dependence of the sheet resistance on the applied magnetic field *H* for InSb and InSb|Pt at 25 K. As shown in Fig. 2(a), the sheet resistance of InSb increses with magnetic fields almost linearly^3^. At 9 T, the ratio of the magnetoresistance 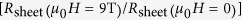 is over 3000%, and the resistance value of InSb surpasses that of Pt at 9 T. This very large magnetoresistance observed in InSb affects the magnetoresistance effect in InSb|Pt significantly. For InSb|Pt, as shown in Fig. 2(a), the magnetic field dependence of the sheet resistance is clearly different from that for InSb. The sheet resistance in InSb|Pt increases rapidly in a low magnetic-field regime, but tends to saturate at high magnetic fields. The sheet resistance of InSb|Pt at 9 T is about 25 Ω, which is almost the same as that of the Pt film [Fig. 1(c)]. In InSb|Pt, as the magnetic field is increased, dominant conduction changes from InSb to Pt due to the very large magnetoresisntace in InSb [see also Fig. 2(c)].

The crossover of dominant conduction from InSb to Pt under strong magnetic fields produces a non-monotonic magnetic field dependence of the Hall resistance in InSb|Pt. In Fig. 2(b), the magnetic field (*H*) dependence of the Hall resistance 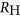 is shown for InSb and InSb|Pt at the same temperature, 25 K. The Hall resistance in InSb|Pt first increases in the magnitude, but shows a peak structure around 1 T, followed by gradual decrease with increasing magnetic fields. This decrease at high magnetic fields is almost proportional to 1/*H* and seems to converge to zero in the high-field limit. The observed magnetic field dependence is not explained by two carrier (*i.e.* electrons and holes) transport effects^13^, since the Hall resistance shows no sign change under strong magnetic fields. Instead, the overall magnetic-field dependence is reproduced practically by a dispersion-type function 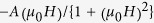 (*A*: a constant), as shown by the solid curve in Fig. 2(b). This dispersion-like Hall resistance is not observed in InSb, where the Hall resistance increases just in proportion to magnetic fields.

**Figure 3:**
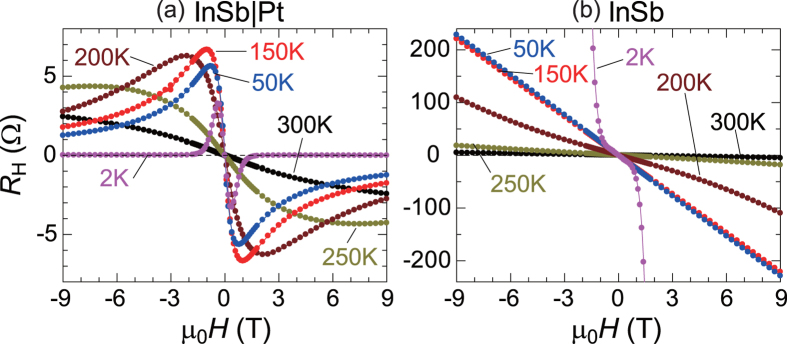
Hall resistance at various temperatures. Magnetic field (*H*) dependence of the Hall resistance 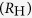 for (**a**) InSb|Pt and (**b**) InSb at various temperatures.

The Hall resistance in InSb|Pt at various temperatures is shown as a function of magnetic fields in Fig. 3(a). At 300 K, the Hall resistance in InSb|Pt is almost proportional to magnetic fields, since the magnetoresistance effect of InSb at 300 K is so small that the electric conduction of InSb remains dominant even at high magnetic fields [Fig. S1(a)]. With decreasing temperature, however, the Hall coefficient of InSb increases largely, as shown in Fig. 3(b). Corresponding to this increase in the Hall resistance, the ratio of the magnetoresistance also increases at low temperatures [Fig. S1(a)], and thus the change in dominant conduction from InSb to Pt with increasing magnetic field strength becomes more significant at lower temperatures. In fact, as shown in Fig. 3(a), the dispersion-type magnetic field dependence of the Hall resistance is observed in InSb|Pt below 200 K [see also Fig. S2]. At 2 K, the magnetic freeze-out effect^11^,^14^,^15^ occurs around 1 T for InSb [Fig. 3(b)]. Because of this localization effect of electrons, the Hall resistance exhibits very small values above 1 T at 2 K for InSb|Pt in Fig. 3(a).

**Figure 4:**
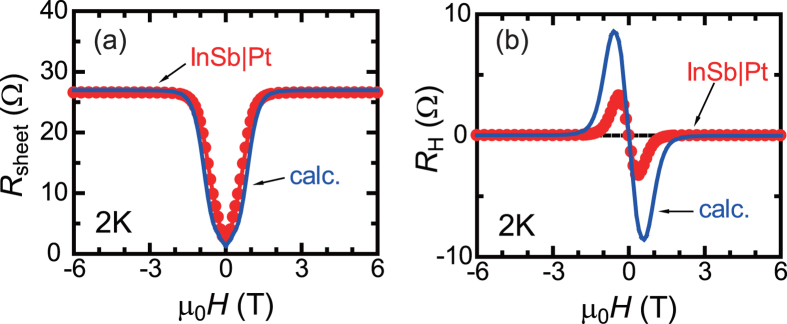
Comparison of calculations with experiments. Magnetic field (*H*) dependence of (**a**) magnetoresistance 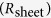 and (**b**) Hall resistance 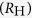 for InSb|Pt (circle, red), compared with the curves (blue) calculated using eq. (1) and eq. (2).

To discuss the magnetic field dependence of magnetoresistance and Hall resistance in InSb|Pt quantitatively, let us consider a parallel circuit model as illustrated in Fig. 1(d). When an electric current is applied to InSb|Pt, electric current flows both in the Pt film and in the InSb substrate. Hence, the total sheet resistance of InSb|Pt is given by


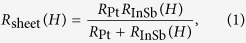


where the sheet resistances of InSb 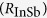 and Pt 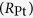 in the zero magnetic field are shown in Fig. 1(c) and magnetoresistance in Pt is neglected in the whole temperature range [see Fig. 2(c)]. In Fig. 4(a), we show a simulated curve of the magnetoresistance for InSb|Pt at 2 K using eq. (1). Since the magnetoresistance effect at high magnetic fields is very large for InSb at 2 K, almost all the electric current passes the Pt film in the high magnetic-field range and the simulated sheet resistance is saturated at 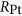 above 2 T. As shown in Fig. 4(a), the overall magnetic field dependence of the sheet resistance is well explained by the parallel circuit model of eq. (1).

Also for the Hall resistance in InSb|Pt, a similar parallel circuit model^16^,^17^ leads to the following relation:


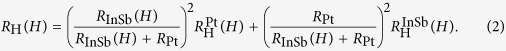


Here, the Hall resistance for Pt and InSb is denoted by 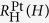 and 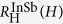, respectively. The Hall coefficient for Pt is 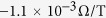 at 300 K and hardly changes with temperature [Fig. 2(c)]. The magnetic-field dependence of the simulated Hall resistance for InSb|Pt at 2 K using eq. (2) is shown in Fig. 4(b). The overall magnetic-field profile of the Hall resistance is reproduced by eq. (2), while the peak values of the Hall resistance are different from the simulated curve. It is noted that the calculated curves using eq. (1) and eq. (2) appreciably deviate from experimental results at high temperatures, indicating that such a simple parallel circuit model is not sufficient to fully explain the experimental results; large deviation of simulated curves from experimental results at 25 K are shown in Fig. S4. The amount of the electric current flowing through the Pt layer seems to be larger than that expected from eq. (1) and eq. (2) under magnetic fields, as indicated by the larger magnetoresistance ratio for InSb|Pt than InSb in low magnetic-field region at 25 K [Fig. 2(a)]. Interface resistance between Pt and InSb need to be incorporated for more detailed analysis. Bending of energy bands at the InSb|Pt interface and their changes with magnetic fields may be also important.

## Discussion

The dispersion-type magnetic field dependence of the Hall resistance was observed for InSb|Pt at low temperatures, *e.g.* 25 K [Fig. 2(b)], 50 K, 100 K, and 150 K [Fig. S2(a–c)]. Such a dispersion-type magnetic field dependence of the Hall resistance is reproduced for another InSb|Pt sample [Fig. S3] in which the InSb wafer was grown in a different group (Chinese Academy of Sciences). This result indicates that the dispersion-like Hall response is not an extrinsic origin, such as magnetic impurities in InSb wafers. As discussed in Fig. 4, the magnetic field dependences of sheet and Hall resistances are almost explained by parallel circuit models, where the crossover of dominant conduction from InSb to Pt with increasing magnetic-field strength is taken into account.

At low magnetic fields where the conduction of InSb is dominant over Pt, eq. (2) is reduced to 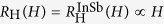. This relation was indeed observed at 25 K in the low magnetic-field range, as shown in Fig. 2(b). At high magnetic fields, by contrast, the second term in eq. (2) reads 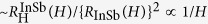. As shown in Fig. S5, the observed magnetic field dependence of the Hall resistance at high magnetic fields supports this expectation from eq. (2). The *H* and 1/*H* dependences observed at low and high magnetic fields respectively are consistent with a dispersion-type function, 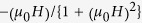, which practically reproduces the overall magnetic field dependence of the Hall resistance for InSb|Pt at low temperatures [Figs 2(b) and S2(a–c)].

In the Boltzmann transport theory, the Hall *conductance* can show a dispersion-like magnetic-field dependence in conductors with ultrahigh mobilities. Such a dispersion-resonance profile in the Hall conductance was recently reported for a topological crystalline insulator Pb_1−*x*_Sn_*x*_Se^18^ and a Dirac semimetal Cd_3_As_2_^19^. The dispersion-type profile of the Hall resistance observed here, on the contrary, is not explained by such a conventional transport theory for single materials. The combination of quantum linear magnetoresistance in InSb and metallic conduction with low mobilities in Pt is a key to realize the unconventional magnetic-field profile of the Hall resistance.

## Methods

### Sample preparation

Several parallelpiped-shaped samples 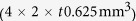 of InSb were cut from a commercial Te-doped (*n*-type) InSb wafer (Wafer Technology Ltd.). The carrier density is 4–9 × 10^14^ cm^−3^. On a polished (100) plane of the InSb substrate, a 7-nm-thick polycrystalline Pt film was deposited by sputtering in an Ar atmosphere at room temperature. Also, for reference, a 7-nm-thick Pt film was grown on an oxidized Si substrate at the same time. The thickness of the Pt films was determined by X-ray reflectivity measurements.

### Magneto-transport measurements

In the measurements of magnetoresistance and Hall resistance for InSb and InSb|Pt, an electric current was applied along a [0, 1, 1] direction, and an external magnetic field along [1, 0, 0] which is perpendicular to the sample plane. Voltage electrodes for the measurement of longitudinal and Hall resistances were made on the Pt film using a Ag paste. The measurements were performed at low temperatures down to 2 K in a Physical Property Measurement System (Quantum Design, Inc.).

## Additional Information

**How to cite this article**: Shiomi, Y. and Saitoh, E. Dispersion-type Hall resistance in InSb|Pt hybrid systems. *Sci. Rep.* **6**, 22085; doi: 10.1038/srep22085 (2016).

## Supplementary Material

Supplementary Information
