# Supplementary material for: Dispersion-type Hall resistance in InSb|Pt hybrid systems
Source: Sci Rep. 2016 Feb 24;6:22085. doi: 10.1038/srep22085 (PMC4764982; doi:10.1038/srep22085)
Supplement: Supplementary Information [file srep22085-s1.pdf]

Supplementary Information for "Dispersion-type Hall resistance in InSb|Pt hybrid systems"

Y. Shiomi and E. Saitoh

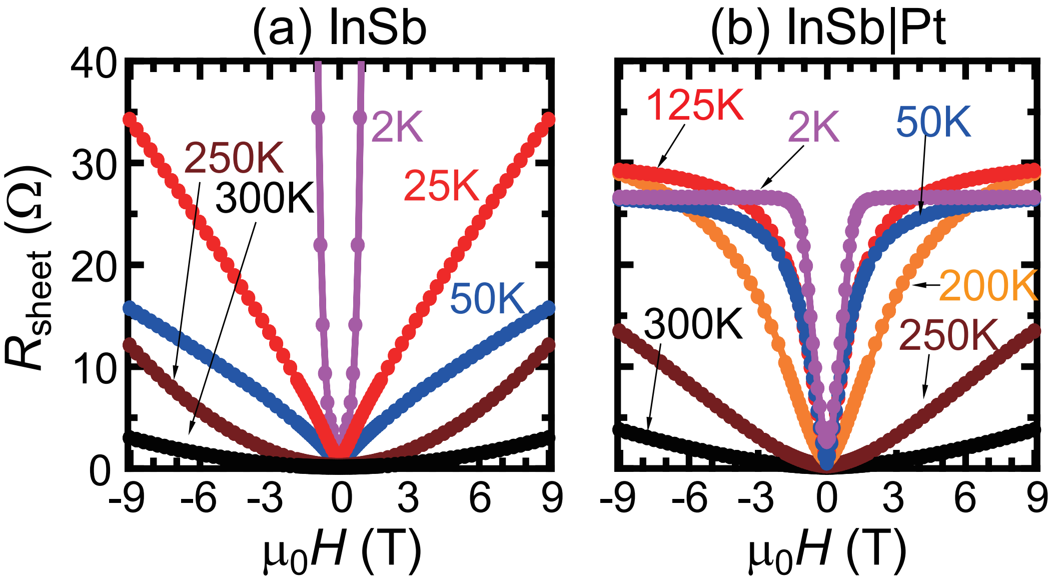

FIG. S1: **Magnetoresistance at various temperatures.** Magnetic field (H) dependence of the sheet resistance (R_{sheet}) for (a) InSb and (b) InSb|Pt at various temperatures.

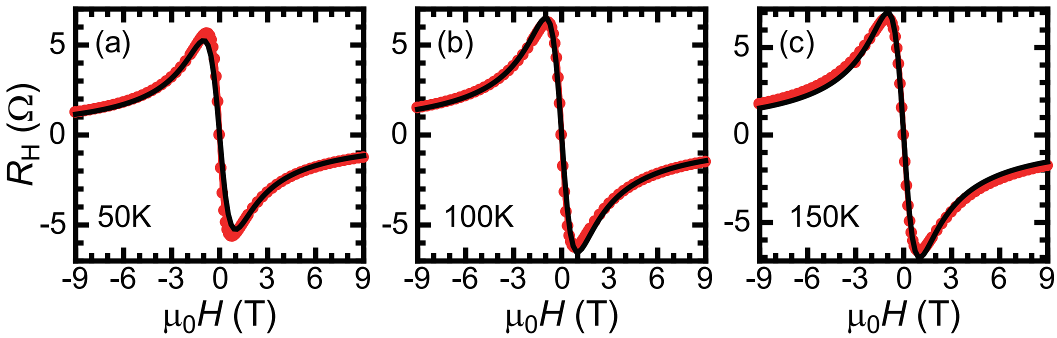

FIG. S2: **Comparison of Hall resistances with dispersion-type functions.** Magnetic field (H) dependence of the Hall resistance (R_{H}) for InSb|Pt at (a) 50 K, (b) 100 K, and (c) 150 K. The experimental results are compared with a dispersion-type function, $-A(\mu_0 H)/\{1 + (\mu_0 H)^2\}$ (A: constant with magnetic fields) at each temperature (black curve).

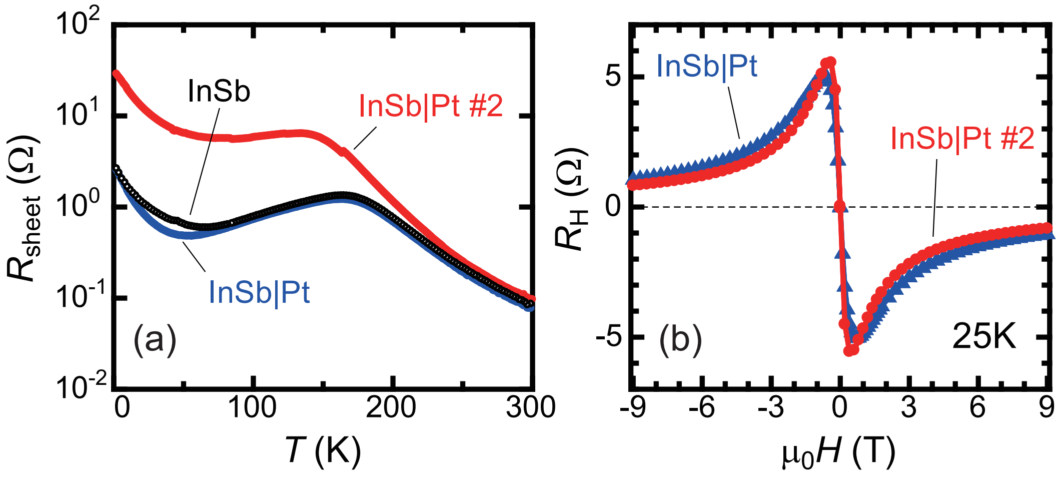

FIG. S3: **Reproducibility for another InSb substrate.** (a) Temperature (T) dependence of the sheet resistance ($R_{\rm sheet}$) for another InSb|Pt sample (denoted by InSb|Pt #2), where the InSb (100) wafer [n-type (Te-doped), the carrier concentration of 3-6$\times 10^{14}$ cm^{-3}] was grown in Chinese Academy of Sciences. (b) Magnetic field (H) dependence of the Hall resistance ($R_{\rm H}$) for InSb|Pt #2 at 25 K. In (a) and (b), the experimental results for InSb and InSb|Pt shown in the main text are also presented for reference.

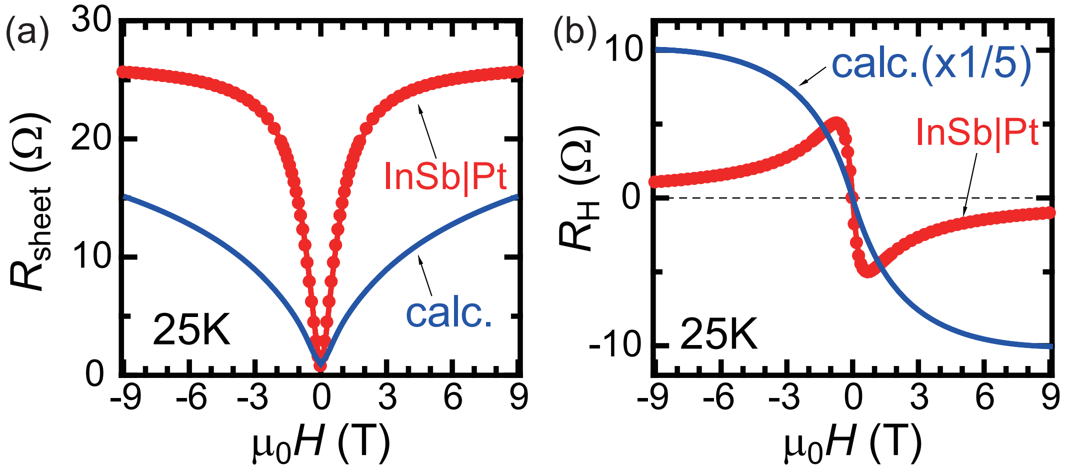

FIG. S4: **Experimental data and calculation results at** 25 **K.** Magnetic field (H) dependence of (a) the sheet resistance ($R_{\rm sheet}$) and (b) the Hall resistance ($R_{\rm H}$) for InSb|Pt at 25 K. The experimental data are compared with curves calculated using eq. (1) and eq. (2) at 25 K. Since the amount of the electric current flowing through the Pt layer is larger than that expected from eq. (1) and eq. (2) under external magnetic fields, the experimental results largely deviate from the calculation results.

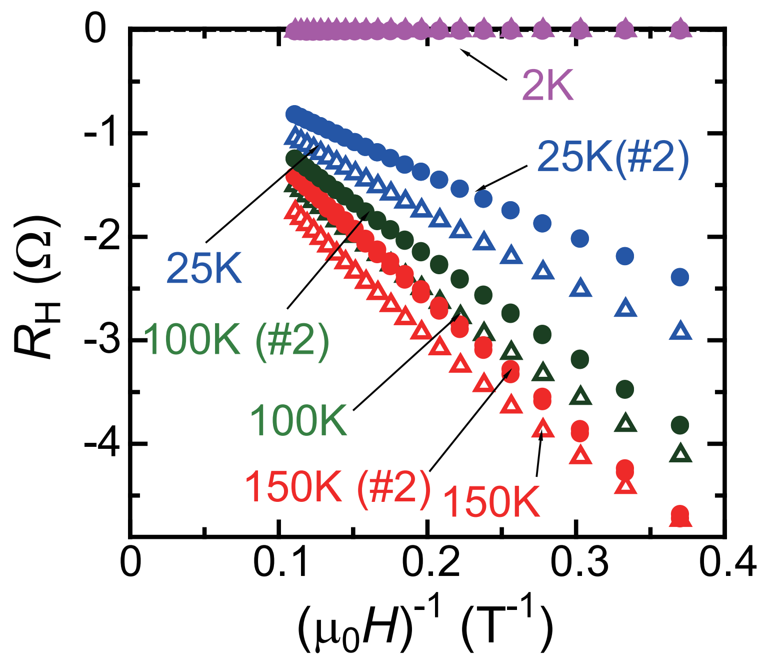

FIG. S5: **Hall resistances plotted against the inverse of magnetic fields.** The Hall resistance (R_H) as a function of the inverse of magnetic fields [$1/(\mu_0 H)$] at high magnetic fields for two InSb|Pt samples.
